# ‘Gender is not even a side issue…it’s a non-issue’: career trajectories and experiences from the perspective of male and female healthcare managers in Kenya

**DOI:** 10.1093/heapol/czz019

**Published:** 2019-04-25

**Authors:** Kelly W Muraya, Veloshnee Govender, Chinyere Mbachu, Nkoli P Uguru, Sassy Molyneux

**Affiliations:** 1 Health Systems Research Department, KEMRI-Wellcome Trust Research Programme, Nairobi, Kenya; 2 Department for Reproductive Health and Research, World Health Organization, Avenue Appia 20, Geneva 1211, Switzerland; 3 Department of Community Medicine, University of Nigeria, Nsukka Road, Nsukka, Nigeria; 4 Department of Preventive Dentistry, College of Medicine, University of Nigeria, Nsukka Road, Nsukka, Nigeria; 5 Nuffield Department of Medicine, Centre for Tropical Medicine and Global Health, University of Oxford, Old Road Campus, Oxford, Headington, UK

**Keywords:** Gender and health systems, health managers, health leadership, health governance, LMICs, Kenya

## Abstract

Women comprise a significant proportion of the health workforce globally but remain under-represented in the higher professional categories. Concern about the under-representation of women in health leadership positions has resulted in increased research on the topic, although this research has focused primarily on high-income countries. An improved understanding of the career trajectories and experiences of healthcare leaders in low- and middle-income countries (LMICs), and the role of gender, is therefore needed. This qualitative case study was undertaken in two counties in coastal Kenya. Drawing on the life-history approach, 12 male and 13 female healthcare leaders were interviewed between August 2015 and July 2016 on their career progression and related experiences. Although gender was not spontaneously identified as a significant influence, closer exploration of responses revealed that gendered factors played an important role. Most fundamentally, women’s role as child bearers and gendered societal expectations including child nurturing and other domestic responsibilities can influence their ability to take up leadership opportunities, and their selection and appointment as leaders. Women’s selection and appointment as leaders may also be influenced by positive discrimination policies (in favour of women), and by perceptions of women and men as having different leadership styles (against women, who some described as more emotive and reactive). These gendered influences intersect in relatively invisible ways with other factors more readily identified by respondents to influence their progression and experience. These factors included: professional cadre, with doctors more likely to be selected into leadership roles; and personal and professional support systems ranging from family support and role models, through to professional mentorship and continuing education. We discuss the implications of these findings for policy, practice and research, including highlighting the need for more in-depth intersectionality analyses of leadership experience in LMICs.

**Key Messages**

- Gender issues were not directly identified as a significant influence on men or women’s health leadership progression. However, the important influence of gender roles and relations emerged in men and women’s different priorities, opportunities and concerns.
- Beyond women’s role as child bearers and gendered societal expectations including child nurturing and other domestic responsibilities, women’s selection and appointment as leaders also appeared to have been influenced by positive discrimination policies and gendered views of leadership style.
- These gendered influences intersect in relatively invisible ways with other factors much more readily identified by respondents to influence their career progression and experience, including professional cadre and personal and professional support systems such as family support, role models, professional mentorship and continuing education.
- More in-depth intersectionality analyses of leadership experience in LMICs are urgently needed.

## Introduction

Strong leadership and management are crucial for building robust health systems that are responsive to population needs [Hogan *et al.*, 1994; Conger and Kanungo, 1998; Lord and Maher, 2002; International Labour Organization (ILO), 2012; Daire *et al.*, 2014]. Effective leadership and management are especially critical in low- and middle-income country (LMIC) settings where health systems face complex challenges including fragility, resource scarcity and high disease burdens. Although leadership and management are two discrete concepts in theory, they overlap significantly in practice and can be conceptualized as important components of the same job (Daire *et al.*, 2014). Some authors argue that in the context of the health sector it might be more useful to approach the two concepts as inter-related and overlapping, and that the notion of ‘managers who lead’ offers a more holistic approach to health policy planning and implementation, and strengthening of organizational capacity (Galer *et al.*, 2005; Daire *et al.*, 2014). Leadership and management were therefore considered as two inter-related concepts in this study.

Despite increased efforts at global, regional and country level to promote gender equality and women’s empowerment, women continue to be under-represented in leadership positions across a range of sectors and geographic regions (ILO, 2012). Concern about this under-representation of women in leadership and managerial positions has resulted in increased research interest around gender and leadership (ILO, 2012). However, much of this research has been conducted in corporate and high-income settings (Eagly and Karau, 2002; Hatcher, 2003; Foundation of the American College of Healthcare Executives, 2006; Sanchez-Hucles and Davis, 2010; Koenig *et al.*, 2011; Minelgaite Snaebjornsson and Edvardsson, 2012; Katila and Eriksson, 2013; Askehave and Zethsen, 2014; Sabharwal, 2015).

In the health sector specifically, women comprise a substantial proportion of the global health workforce (Hoss *et al.*, 2011). They are, however, over-represented in lower-paying, lower-status occupations and their representation declines with respect to higher professional categories including managerial and decision-making positions (World Health Organization, 2008; Downs *et al.*, 2014). The role of gender in healthcare leadership in LMICs nevertheless remains under-researched, with existing research on leadership largely focusing on the private healthcare industry in the USA (Foundation of the American College of Healthcare Executives, 2006; Lantz, 2008; Branin, 2009). We identified only one study focusing on the role of gender in health leadership in a middle-income country (Tlaiss, 2013); a study undertaken in Lebanon to explore impediments and enablers of Middle Eastern women’s career advancement in the health sector. This study showed that prejudicial cultural values and gendered social roles and expectations hindered the career advancement of women in this context. It also highlighted how women’s agency at an individual level acted as an enabler in navigating meso- and macro-level factors such as patriarchal cultural norms and intrinsic discrimination in their organizations; supporting their career moves upwards and their negotiation into management positions (Tlaiss, 2013). Given the paucity of data on gender and leadership in healthcare settings in LMICs, it is important to build an evidence base of the needs, experiences and expectations of healthcare leaders and situate these in broader social, political and institutional contexts. This will increase understanding of the requirements of healthcare leaders, and potentially contribute to shaping and informing policy and practice in relation to healthcare leadership development, training and support.

In Kenya where this study was conducted, the country moved from a centralized system of governance to a devolved system following the signing of a new constitution in 2010—which became operational in 2013 (National Council for Law Reporting (Kenya Law), 2018a); with a somewhat ‘turbulent’ transition period in the first few years fraught with political tensions between the national and county governments. Devolution resulted in 47 semi-autonomous counties and the decentralization of many functions to county level, including some health functions. Consequently, elected county leaders now have the authority to appoint local county cabinets that oversee county functions including health-related functions (National Council for Law Reporting (Kenya Law), 2018a). These local elected and appointed leaders also play a key role in setting county health priorities, allocating resources received from the national level, and undertaking various forms of local resource mobilization to strengthen service provision (National Council for Law Reporting (Kenya Law), 2018a), highlighting the crucial role of governance and leadership in the health sector. The 2010 Constitution also makes specific reference to gender representation in both appointed and elective leadership bodies, in turn directly impacting on diversity and human resource policies within the public service (Public Service Commission, 2016; National Council for Law Reporting (Kenya Law), 2018a). In this context of devolution, this study aimed to describe and explore career pathways and leadership experiences of healthcare managers at sub-national level in Kenya using a gender lens.

## Methods

### Study setting and design

This exploratory qualitative case study (Yin, 1994; Creswell, 1998) was undertaken in coastal Kenya. Two case study counties—Mombasa (urban) and Kilifi (semi-rural)—were selected primarily to build on existing health governance research work. Each of the two counties are divided into sub-counties with corresponding Sub-County Health Management Teams (SCHMTs). Mombasa has four SCHMTs namely Mvita, Changamwe, Kisauni and Likoni; whereas Kilifi has three SCHMTs—Kilifi, Malindi and Kaloleni. Respondents were drawn from both the county level (4) and the various SCHMTs (21), totalling 25 respondents. In addition to ensuring gender balance, respondents were selected to reflect varying length of experience in the health sector and the diversity of managerial categories within both the county and sub-county levels. Data for this work were collected between August 2015 and June 2016, only 2 years post the initiation of devolution in Kenya. Drawing on the life-history approach (Lewis, 2008), in-depth individual interviews that lasted between 60 and 90 min were conducted with all respondents. All interviews were led by the primary author (KWM), a postdoctoral social scientist with extensive experience in conducting interviews specifically, and undertaking qualitative research more broadly. The life-history approach allowed for in-depth exploration of participants’ backgrounds, career trajectories and experiences. Broad topics covered included: An in-depth discussion of respondents’ family and personal backgrounds, motivation for joining the health sector and career trajectories dating back to their initial training and beyond; their job roles and responsibilities including positive highlights and challenges; and their overall experience and perception of leadership. All interviews were audio-recorded and transcribed verbatim. The principal investigator also maintained a reflective field diary documenting each of the interviews and noting any important observations.

### Summary of respondents

Of the 25 respondents, 13 were female and 12 were male. Table 1 below summarizes some characteristics of the study respondents. Health manager cadres included county level leaders (health executives, directors of health, chief officers of health), and sub-county leaders (medical officers of health, public health nurses, facility management nurses, public health officers, disease surveillance co-ordinators and programme co-ordinators).

**Table 1:** Summary characteristics of study respondents

Code	Sex	Background/training	Commencement of service in the health sector
Kilifi county			
R001	Male	Clinical medicine (higher diploma level)	2013
R002	Female	Nursing	1995
R003	Male	Pharmacy	2008
R004	Male	Clinical medicine (higher diploma level)	1984
R005	Male	Environmental health sciences and public health	1993
R006	Female	Nursing	1995
R007	Female	Health information systems	1998
R008	Male	Pharmacy	2006
R009	Female	Nursing	2010
R010	Male	Nursing	Late 1980s
R011	Female	Environmental health science and public health	1990
R012	Male	Nursing	Early 1990s
Mombasa county			
R013	Female	Commerce and marketing	2013
R014	Male	Medicine (bachelor’s degree)	Late 1980s
R015	Female	Dentistry	2000
R016	Female	Pharmacy	Mid-2000s
R017	Male	Pharmacy	Mid-2000s
R018	Female	Nursing	1986
R019	Female	Nursing	2003
R020	Female	Environmental health sciences	1997
R021	Female	Nursing	1985
R022	Female	Public health	1997
R023	Male	Medicine (bachelor’s degree)	2011
R024	Male	Nursing	1992
R025	Male	Public health	Late 1980s

### Data analysis

Data were managed using Nvivo 10 and analysed using thematic content analysis. After in-depth immersion in the data and familiarization with the transcripts, summary tables were developed for each interview. A coding framework based on the research questions and objectives, as well as from preliminary emergent themes was developed through a consultative process engaging the entire research team. Data were then split into codes and rearranged according to thematic content.

### Ethics

Ethical approval for this study was obtained from the primary author’s institute. Prior to data collection, information sessions were conducted with relevant stakeholders at both county and sub-county level to sensitize them on the study. Written informed consent was obtained from all study participants including specific information to audio-record interviews. For participant anonymity and confidentiality, all identifiers have been replaced with numeric codes. All illustrative quotes have also been carefully reviewed for their potential to reveal individual identities.

### Study limitations

One key limitation of this work was the under-representation of respondents from the county level (4), compared with those from the sub-county level (21). This was not by design but rather the result of the busyness and consequent unavailability of county-level officials. Nonetheless, the four respondents from the county level gave rich and insightful information on issues related to experiences and progression in health leadership at the sub-national level. Also, this study focused on individuals who had already attained leadership positions within the health system. Given the repeated emergence of challenges related to promotions and career advancement even within this group, it is likely that views from other categories, e.g. those who have been unsuccessful in securing leadership positions, might have elicited different findings. Finally, in reflecting on the positionality of the primary author as a female interviewer engaging with both male and female (mostly older) respondents, it is plausible that some individuals might have refrained from discussing or being explicit about certain gender-sensitive topics, or might have over-emphasized other issues such as positive views around female leadership.

## Results

Gender did not spontaneously or explicitly emerge as an influencing factor for any of the respondents in discussions of career trajectories or leadership experience; and there were no obvious comparable differences by sex in the facilitators and barriers discussed by both men and women. Importantly however, implicit influences of gender and other social hierarchies emerged in discussions. This section begins by presenting an overview of career trajectories across all participants including their motivation for joining the health sector and perspectives of key enablers and constraints. The less visible gendered influences that we suggest play a role in leadership progression and experience are then discussed.

### Career trajectories

Career paths and trajectories were varied with most respondents taking on further studies such as additional certificate, diploma and masters level courses while already in-service to augment their professional and educational qualifications. These additional qualifications were perceived as an added advantage with regards to general career progression, particularly in the context of limited opportunities for promotion. Many of the respondents had always intended to work in health professions. The reasons for this were diverse including ‘altruistic’ reasons such as the desire to help the less fortunate and improve the (health) status of their communities, or having experienced prolonged illness of a family member often with poor access to quality healthcare which in turn led to a desire to assist others in a similar position.

*What really motivated me is my mother. She was sick, I used to take her to see a consultant. Every day we’d go there and used to wait for two, three, four hours. So from that time I said*, *‘maybe I should do something that will help other people…At least they will not come and stay for several hours’. So that motivated me to do medicine, at least to help, and especially those people who are less fortunate* (R023, male manager).

Other stated reasons for joining the health sector included influence from friends and family members who were already working within health professions, and pragmatic reasons such as affordability of health-related training courses post-secondary schooling and availability of related jobs. There were, however, a few individuals who had never intended to work in the health sector but ended up in health professions due to family pressure or perceived lack of alternatives.

After formal entry into the health sector, many of the respondents then progressed through the system—usually gradually over time and sometimes more abruptly—to various health leadership and managerial positions. Progression to these positions was either voluntary or involuntary and respondents had varied views about their advancement to leadership positions. All but one of the respondents in this study would be regarded as hybrid managers (Gronn, 2009). That is, trained health professionals who are required to take on more managerial and administrative responsibilities as well as maintain some of their original professional responsibilities. There were two categories of respondents in this regard. There were those who, despite having been (involuntarily) selected to take on managerial roles, were content and willing to take up more management roles at the expense of their technical/clinical practice. On the other hand, some respondents would have preferred to focus on their practice and had reluctantly taken up their managerial positions. Below are two quotes to illustrate these two categories. The first quote illustrates a ‘willing manager’, whereas the second quote illustrates a more ‘reluctant manager’ who had ended up in management more by default than choice.

*…when I got an opportunity to get into management for health, I think I liked the management bit better…Management is something that just came with this [position], but I’m really enjoying the experience, and also the challenges and successes that come with it* (R003, male manager).

*As doctors I don’t think we are trained to be managers…I can give my own example. I was [abruptly] inducted into health leadership, very unfortunate…you might mess up. For me it was learning on the job. This [management] is something that actually nobody has prepared you for…To me that really doesn’t work. Maybe [the person] is good with patients, perhaps maybe good with surgery, but it doesn’t mean that they will actually be good with the leadership…* (R014, male manager).

Related to this was a sense that better mentorship and capacity building right from pre-service training was essential to equip individuals with the necessary skills to take up managerial and leadership positions.

### Enablers of career progression

Regardless of whether they were willing or reluctant managers, both female and male respondents cited a range of common factors at the personal and professional level that enabled them to advance in their careers. Personal enabling factors included: general family support and encouragement including supportive parents and spouses (the latter being given more emphasis by women than men but mentioned by all respondents); influential role models and personal mentors; intrinsic factors such as self-determination and self-discipline; passion, commitment and ambition; and well-wishers who provided financial support for continuing education especially for those respondents who came from less-privileged backgrounds. The quote below illustrates family support and encouragement as an enabling factor:

*…apart from my supportive husband, I also have my parents. My father is also somebody who has really encouraged me to go further since I was a little girl…he knew that we can go higher than where we were. So after he had educated us to that level, you feel challenged, like I should go beyond his vision. So he has really been an inspiration to go further* (R006, female manager).

Professional enabling factors included: professional mentorship; flexible work environments that accommodated for (paid) study leave hence enabling further study while in-service, which in turn increased opportunities for job promotions; partial study scholarships from employers; continuing professional development including on-the-job trainings; and supportive superiors.

### Constraints to career progression

Similarly, there were various personal and professional constraints that were cited as hindering career progression. At the personal level, the key constraints were time and financial limitations. This was particularly noted by women in the context of competing interests such as family obligations, which impeded the ability to take up certain job positions or further study, hence limiting opportunities for career progression.

*Some of the challenges especially being a family woman, it has not been very easy…you want to [further your education], money is needed for school fees for yourself and for the children, so you give the children an upper hand and sometimes you slow down…you have to balance between the family life and the career* (R011, female manager).

The key cited professional constraints to career progression included limited opportunities for promotion as there were many eligible staff and few positions available for advancement. Also noted as constraints by male and female managers were perceived biased promotions based on favouritism (including nepotism), corruption and having the right connections. The quote below illustrates career stagnation due to perceived biased promotions.

*We can’t blame all of our stagnation on the county [devolution], because county adopted us from the national government. And in the national government there was a lot of things, mambo mengi ya kichinichini [corruption], you must be known for you to be promoted, or you must know somebody, you know? They were not following the right criteria, the right process for promoting people* (R019, female manager).

### Role of professional hierarchies

Professional categories were widely viewed by men and women as playing a very dominant role in both appointment to health leadership positions and general career progression. Specifically, medical doctors were stated as being preferentially selected for leadership positions and having faster and clearer career progression pathways than other health workers. This sometimes resulted in tensions between medical doctors and other categories of health workers.

*…there are some cadres, the doctor cadre, it’s taken like a special cadre. You will find that their progression is faster than all these other cadres despite having similar qualifications…you could even be more learned than them, but you find [the system] favours the doctors. The other cadres have been left behind…like my son who is just doing his [medical] internship started in the same job group that I have worked in for the last 25 years. He found me with this career, I gave birth to him, I sent him to school, now he is a doctor and he has entered the same job group as me…Right from the beginning [the system] favours them* (R011, female manager).

### Gender and health leadership

As previously stated, gender was not spontaneously or explicitly perceived as an issue impacting on career progression and leadership experience, and gender-specific or targeted initiatives were not raised by either male or female respondents. In fact, in the broader context of health leadership, gender was perceived by both men and women as a ‘non-issue’.

*[Gender] is not even a side issue…it’s a non-issue. Both our CECs [county executives for health] so far have been ladies…I do not think it’s an issue for our department. If you look for example at the balance of our [management] team, we have about 5-6 females from a team of about seventeen* (R005, male manager).

However, was gender really a non-issue? Indeed, even the suggestion that having approximately one-third representation of one sex within health management bodies means gender is a non-issue is problematic, as it infers that gender equity is solely about having a certain proportion of each sex represented. On further exploration and discussion, some gendered nuances related especially to women’s career progression and perceptions of female leadership styles (which could potentially impact on leadership appointment) emerged. In particular, the role of women as child bearers and nurturers was perceived by both male and female respondents as being disadvantageous to their career progression and ability to take up leadership positions.

*[When appointing a health manager] …if she is female, you have to consider if she has kids or not. That makes a difference. You will find that you select someone, train them and invest so much in them, then after working for only a few months they fall pregnant and go off on maternity leave. Also, once they have a child, the women tend to become irregular with work, there isn’t that commitment…* (R016, female manager).

Additionally, concerns around maintaining a work–life balance were primarily raised by female respondents, with many stating that they sometimes struggled to juggle between full-time work and domestic responsibilities. This was often pegged to cultural and societal expectations of the role of women (and men), and was further exacerbated by the fact that several of the female respondents were also undertaking part-time studies to augment their educational qualifications. All of these potentially impacted on their ability and willingness to take up certain job positions and subsequently their career advancement. As noted above, many of the female respondents stated that having good support systems at home—usually supportive husbands and/or domestic help—assisted them in coping with the demands of their work and personal lives, and thus facilitated their career progression. This suggests that women without such support may not have had the same opportunities to progress.

Although not directly related to career progression, perceptions around male and female leadership styles could also potentially influence leadership selection and appointments. There were complex views on differences between men and women’s character traits and thus leadership styles. Although in general women were perceived as being more honest and able to get more done, both male and female respondents described women as being emotional and reactive in their leadership style in comparison to male leaders who were perceived as more calm and level-headed.

*…men as much as whatever stress they are in, they don’t express it the way women do…We tend to have more outbursts than men. I guess it’s just the way women are made, that whatever stress that you are going through you tend to express it…you can start shouting, you get grumpy, you can start crying…It definitely affects leadership…I have seen it even with our leaders [here]* (R021, female manager).

Additionally, there were male respondents who felt that because women have previously been marginalized with respect to health (and other) leadership positions, when given an opportunity to lead they are necessarily forceful and authoritative in their leadership style as they feel they must ‘prove their ability’.

*This is a society where women have been depressed for a long time. This leadership has very much been a male domain unless something like nursing…Therefore at times, because of asserting their authority, the women go to the extreme. They end up being dictators…Women want to prove that they can be leaders…even to include here where we are…many of the women who have access to leadership positions right away, because it was not a normalcy for women to climb up the ladder to that level, they want to be known that they are the leaders* (R004, male manager).

There were, however, male and female respondents who felt that generally women are better leaders since they are more innovative, and societal roles and expectations inadvertently equip them with the ability to multitask and undertake challenges in a calm and sober manner.

## Discussion

Using a gender lens, this study aimed to understand and explore career progression and experiences of healthcare leaders at sub-national level in Kenya. Twenty-five healthcare managers with almost equal representation of male and female respondents, from two case study counties—Kilifi and Mombasa—were interviewed.

Although career journeys and experiences were varied for both male and female respondents, there were common overall influences both at the personal and professional level with either positive or adverse consequences. Identified key enablers to upward career progression at the personal level included supportive family contexts, personal ambition, commitment and personal role models. On the professional front these included mentorship and supportive working environments that e.g. enabled continuing education while in-service and therefore increased chances of promotion.

Gender, in and of itself, did not spontaneously or openly emerge as an issue in discussions around healthcare leaders’ career progression and experiences. Indeed, there were respondents who regarded gender as a ‘non-issue’ in this context, sometimes relating this to the current number of women occupying health leadership or managerial positions within the county or sub-county. In these cases, gender equity was perceived as having a specified number of women occupying senior positions within the health system. This perception was perhaps not entirely surprising given that the new Kenyan Constitution (National Council for Law Reporting (Kenya Law), 2018a)—which directly impacts on the public service diversity and human resource policies including of the health sector—makes explicit reference to gender representation in leadership. The Constitution states that: ‘no more than two-thirds of the members of representative bodies in each county government…or county executive committee shall be of the same gender’ [Chapter 11, sections 174 (c) and 191 (1)]. Prior to the promulgation of the 2010 Kenyan Constitution, the Equal Opportunities Bill (2007), directed on the duty to promote gender equality at all levels in all sectors and public institutions including ministries (National Council for Law Reporting (Kenya Law), 2018a, b). It, however, did not provide strategies on how to achieve this, and was not implemented in practice as evidenced by a baseline survey undertaken by the Public Service Commission in 2013–2014. The survey found that the ratio of men to women in the public service stood at 70:30 with the ratio of women at policy-making levels reducing to 23% (Public Service Commission, 2016). There was, therefore, added impetus and active effort to increase the number of women in leadership positions including in the public health sector to meet legislative requirements in the context of the new constitutional dispensation (Public Service Commission, 2016). As such, respondents referred to having a certain proportion of female health leaders within the county and sub-county as attainment of gender balance, potentially diminishing the existence of other gendered influences that may impact on career paths and leadership experience.

Despite the non-emergence of gender as an overt or key issue, our findings show that it was far from irrelevant and had a significant influence on career trajectories and health leadership experience. Most fundamentally, prescribed societal roles of women mean that they are often more likely than men to be having to juggle both work and domestic obligations, with important implications for their ability to take up and manage leadership opportunities. For these reasons, the female leaders we interviewed gave emphasis to the importance of having good personal support systems, such as supportive spouses and domestic help, to manage their various responsibilities and advance in their careers. This influence of societal gender-based responsibilities on career progression and leadership is not unique to the Kenyan context, and has been noted in other LMICs such as Lebanon (Tlaiss, 2013) as well as in high-income settings with much more robust employment equity polices (McDonagh, 2010). For example, research in the USA indicates that despite employment equity policies, gender-based gaps in health leadership persist, and especially for women of colour highlighting the intersection of gender with race (Foundation of the American College of Healthcare Executives, 2006; Lantz, 2008; Branin, 2009). Eagly and Carli, focusing on the USA, described the uneven path of upward progression for women in organizations as a ‘labyrinth’, arising from a complex and varied set of overt and subtle challenges including child care needs, sexism and racism in some contexts (Eagly and Carli, 2007). In exploring the nursing leadership ‘labyrinth’ in the USA, McDonagh identified gender-based barriers such as leadership stereotyping based on societal gender-based beliefs and expectations, sex-based discrimination and women’s domestic responsibilities as impacting on women’s experience of, and hampering their progress towards, leadership (McDonagh, 2010).

As has been observed in studies in high-income settings particularly the USA (Due Billing and Alvesson, 2002; Sanchez-Hucles and Davis, 2010; Karelaia and Guillen, 2011; Koenig *et al.*, 2011; Sabharwal, 2015), men and women in this Kenyan context were also perceived as having differing leadership styles attributed primarily to perceived masculine and feminine characteristics. Women were generally described as being reactive and emotive in their leadership style, or unduly authoritative in an attempt to prove their ability; while men were described as calmer and more sober in their approach. As noted in the American studies, and hinted at in our interviews, such perceptions could inadvertently result in gender-bias in selection and appointment to health leadership positions. This could be an important bias in both selecting those who put themselves forwards for leadership positions, and in who is ‘thrust into’ leadership positions, sometimes reluctantly. The latter was raised as a common concern in our study, and has also been observed in other studies looking at facility level managers in Kenya (Nyikuri *et al.*, 2015; Barasa *et al.*, 2017) and South Africa (Daire and Gilson, 2014). If and how this inter-relates with positive discrimination rules in Kenya and beyond requires more specific examination in future studies, which are currently being planned. Unlike gender, professional categories were reported by all interviewees as playing a very dominant role in career progression. Specifically, medical doctors were viewed as a ‘favoured cadre’ who were preferentially appointed to leadership positions, and whose career progression was well-defined and distinct from other health cadres. Preferential selection of medical doctors could also result in unintended gender inequity in health leadership, given that the medical profession in Kenya was *historically* a male-dominated field, with women mostly being in health professions such as nursing (J Kitulu, personal communication). Indeed, a working paper on the labour market for human resources for health in Kenya by Kiambati *et al.* (2013), showed that men made up 70% of medical officers in the country. Such inequity underscores how gender is shaped by other hierarchies, in this case professional categories. Although there was a sense that this pattern was gradually shifting, it was still viewed as a major issue by many of the respondents potentially ‘crowding out’ obvious recognition of, and discussion around, gender influences.

These findings and the broader literature highlight the need for an intersectional lens in future work. In a recent literature review that we conducted on the intersectionality of gender and health systems’ leadership in LMICs Zeinali *et al.*, unpublished data), we did not find any studies which explicitly used an intersectionality lens. This is an important gap: better understanding of the ways in which patriarchal and other social and political structures are infused into organizational structures, processes and daily life is a priority to inform more equitable policy and practice that tackles and addresses gender and its complex interactions with other social stratifications.

### Policy and practice implications

It is essential that LMICs have strong responsive health systems and this means making the most of the potential leadership talent pool (Dhatt *et al.*, 2017). This study highlights multiple factors that impact on career progression and experiences of male and female healthcare managers, eliciting key influences intersecting with gender. The study has important implications for how we: identify and nurture potential leaders with particular sensitivities for female leadership; support and build capacity of healthcare leaders; build gender-sensitive organizational processes and structures that support all leaders to reach their potential; and develop policy positions to ensure equitable development and distribution of female and male leaders across all levels of the health system.

Specifically, our findings suggest the importance of flexible family-friendly policies and arrangements in health systems to increase opportunities for uptake of leadership positions whilst still managing domestic responsibilities, as well as support with the challenges of balancing family and work-life that were particularly pertinent for women. This is supported by the Public Service Commission Diversity Policy (2016), which establishes strategies for attracting the diverse groups into the public service as well as measures aimed at creating an environment that is conducive and respects diversity. While there are many relevant sections for women’s leadership in health systems in this policy (e.g. 2.3.5 on promotion and career progression and 2.3.6 on training and capacity building), sub-section 2.2.3 on gender diversity makes explicit reference for the need ‘to take necessary measures to provide supporting social services to enable parents to combine family obligations with work responsibilities’ (Public Service Commission, 2016, pp. 12–13). It does not give specific strategies on how this can be achieved, but examples of how such policies and arrangements have been instituted in other settings (particularly high-income countries) and that could be adapted to the Kenyan health system include: flex-time, which is a flexible working schedule that allows individuals to choose when they work as long as they put in the required hours; job-sharing where two or more individuals share a single position and therefore only work a fraction of the required time; temporary or permanent switch to part-time employment; allowing work away from the worksite, e.g. where an employee works from home all or some of the time and on-site day care, particularly where this is free or subsidized (Community Tool Box, University of Kansas, 2018). Specific country settings and leadership roles would, however, need to be carefully considered in adapting and implementing any of these policies.

Beyond family-friendly policies and arrangements, positive professional-level influences can be built upon to enhance career progression and provide supportive working structures. Potential examples include: offering gender-sensitive flexible training programmes that allow trainees to undertake training over a prolonged period of time, and with modules scheduled to fit into existing work and personal life responsibilities; paid study leave for specialist leadership training, and potentially amending job descriptions and rewards to allow for and acknowledge such training; recognition of the important role played by personal support particularly for women, e.g. by allowing people time to do related assignments around their work and personal lives, or encouraging them to undertake assignments that take forward work-related needs thus offering double-value. Whilst these suggested interventions are potentially relevant for all future leaders, it would be important—and even necessary—to be cognizant of the role of gender in leadership progression, and consequently design and implement them in support of having a more gender-balanced health leadership landscape.
